# Supplementary material for: Digital telomere measurement by long-read sequencing distinguishes healthy aging from disease
Source: Nat Commun. 2024 Jun 18;15:5148. doi: 10.1038/s41467-024-49007-4 (PMC11189511; doi:10.1038/s41467-024-49007-4)
Supplement: Supplementary file 1 — Supplementary Information [file 41467_2024_49007_MOESM1_ESM.pdf]

Supplementary Figures

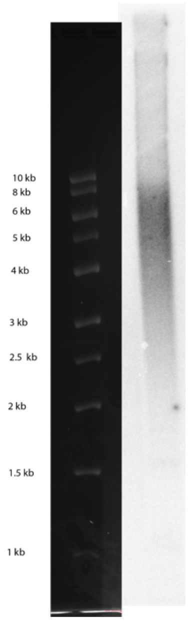

Supplementary Figure 1. Telomere restriction fragment Southern Blot analysis of HEK293T genomic DNA from Figure 1B. Phosphor image of TRF from HEK293T genomic DNA (right) alongside ethidium bromide image of DNA ladder run on the same gel, aligned.

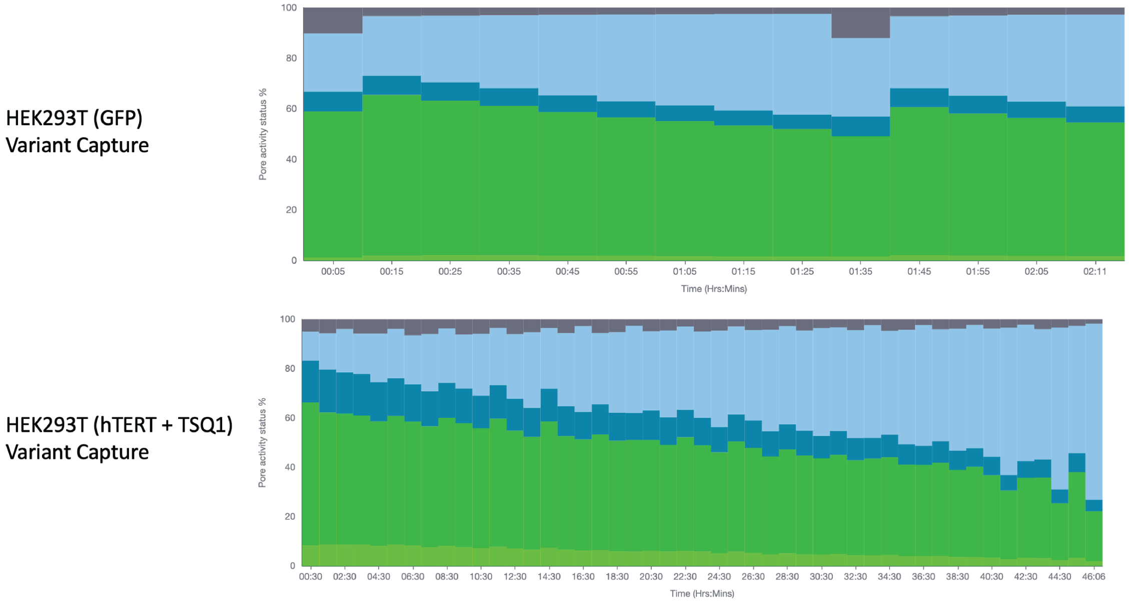

Supplementary Figure 2. Variant telomere capture only produces successful libraries in HEK293T cells transiently transfected with a variant telomerase RNA component template (TSQ1). Pore occupancy vs. time as produced by ONT MinKNOW software during sequencing. Dark green bars represent pores available for sequencing, light green represent actively sequencing pore

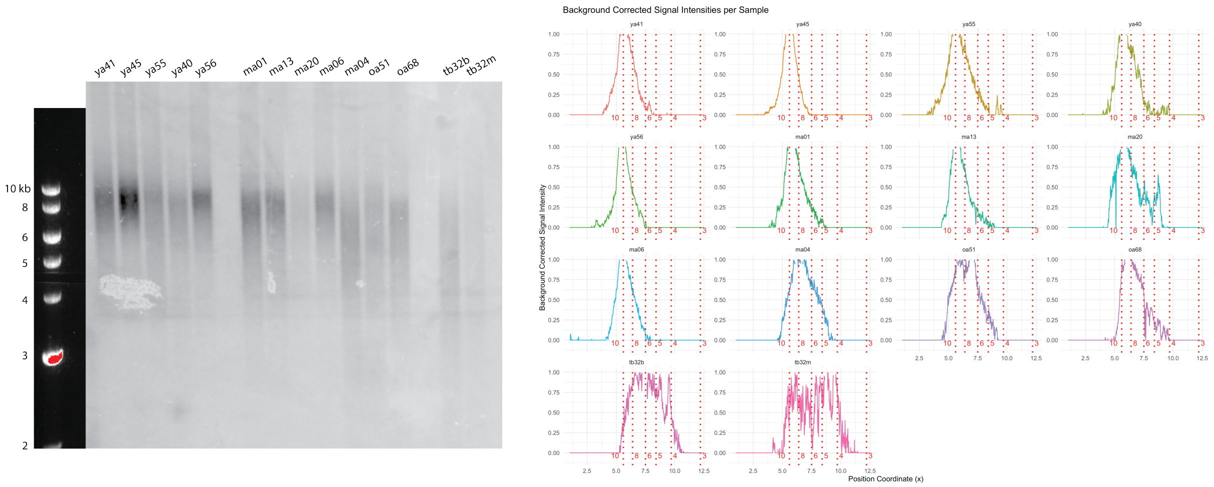

Supplementary Figure 3. Telomere restriction fragment Southern Blot analysis of twelve healthy donors and Stanford Hospital patient. (left) Phosphor image of TRF from twelve healthy donors and Stanford hospital patient aligned to ethidium bromide image of corresponding DNA ladder from gel. (right) Quantitative analysis of signal from TRF Southern Blot using R (4.1.0).

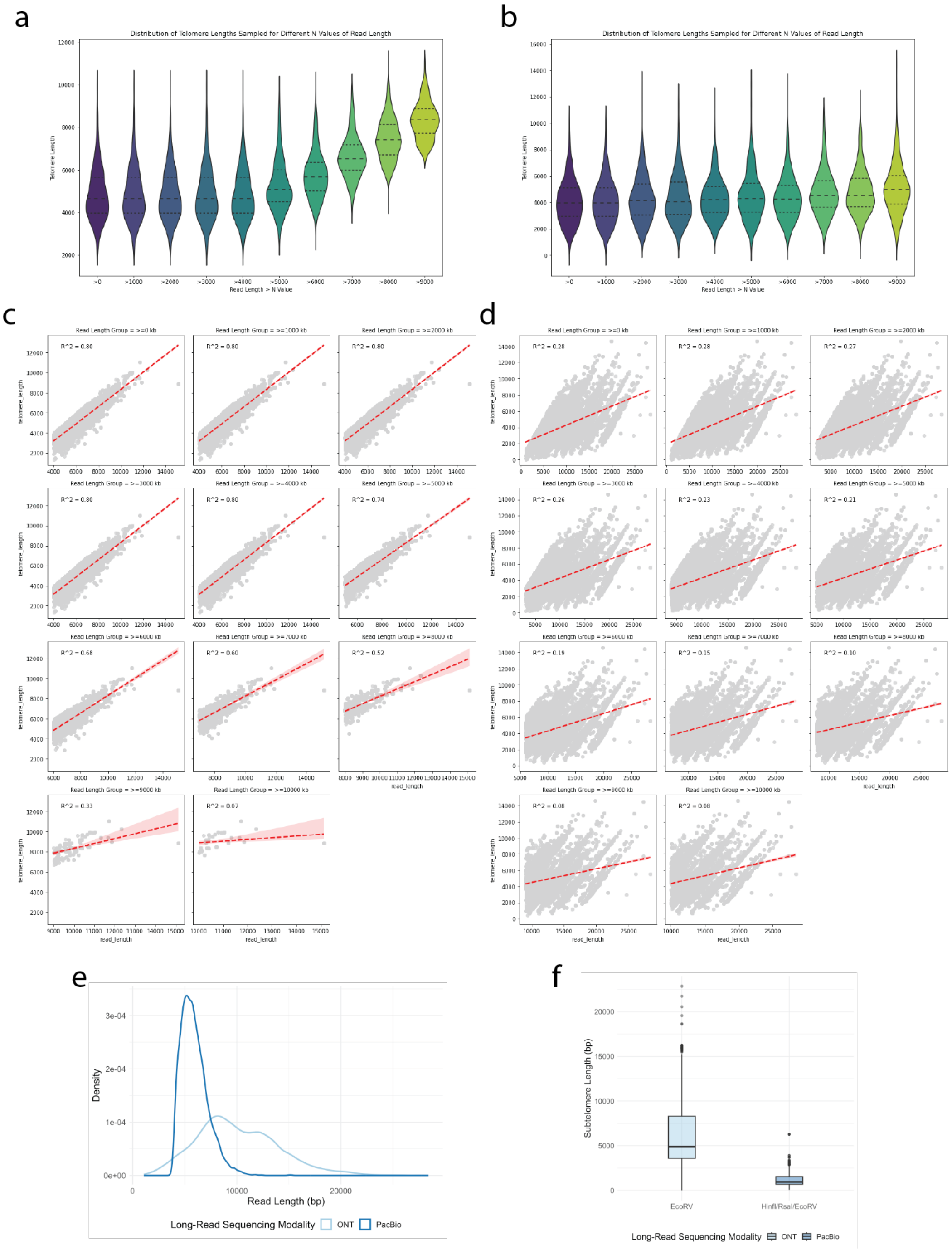

Supplementary Figure 4. PacBio but not Oxford nanopore telomere length measurements are bottle-necked by read length. HEK293T telomere length distributions representing sample of 1000 telomere measurements randomly selected from telomeric reads with a read-length longer than incrementally increasing cutoffs, as measured by PacBio (a, data obtained from *26)* or ONT long-reads (b, original data). Correlation between measured telomere length and read length from PacBio (c, *26*) or ONT (d, original data) long-read sequencing digital telomere length measurement. (e) Read length density distributions for PacBio or ONT data previously demonstrated. (f) Aggregate subtelomere length measured following single or combination restriction digestion in telomeric reads by ONT (left, original data), or PacBio (right, *26*).

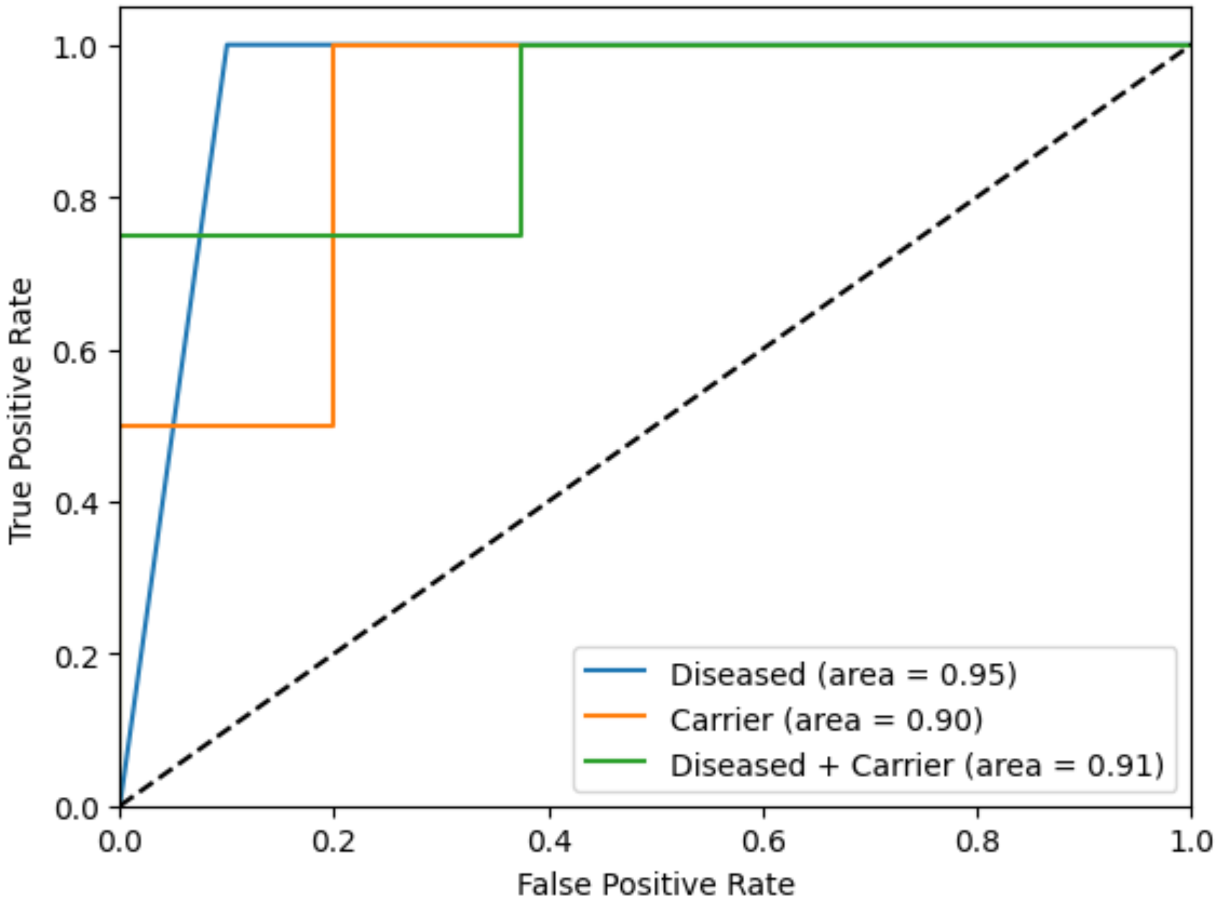

Supplementary Figure 5. Receiver-operator curve for logistic regression binary classification model. Summary statistics from telomere length distributions of 14 healthy individuals and 8 TBD patients produced by Telometer were used as input for three logistic regression binary classification models classifying symptomatic patients versus healthy donors (blue); unaffected carriers versus healthy donors (orange); or both symptomatic patients and unaffected carriers versus healthy donors (green).

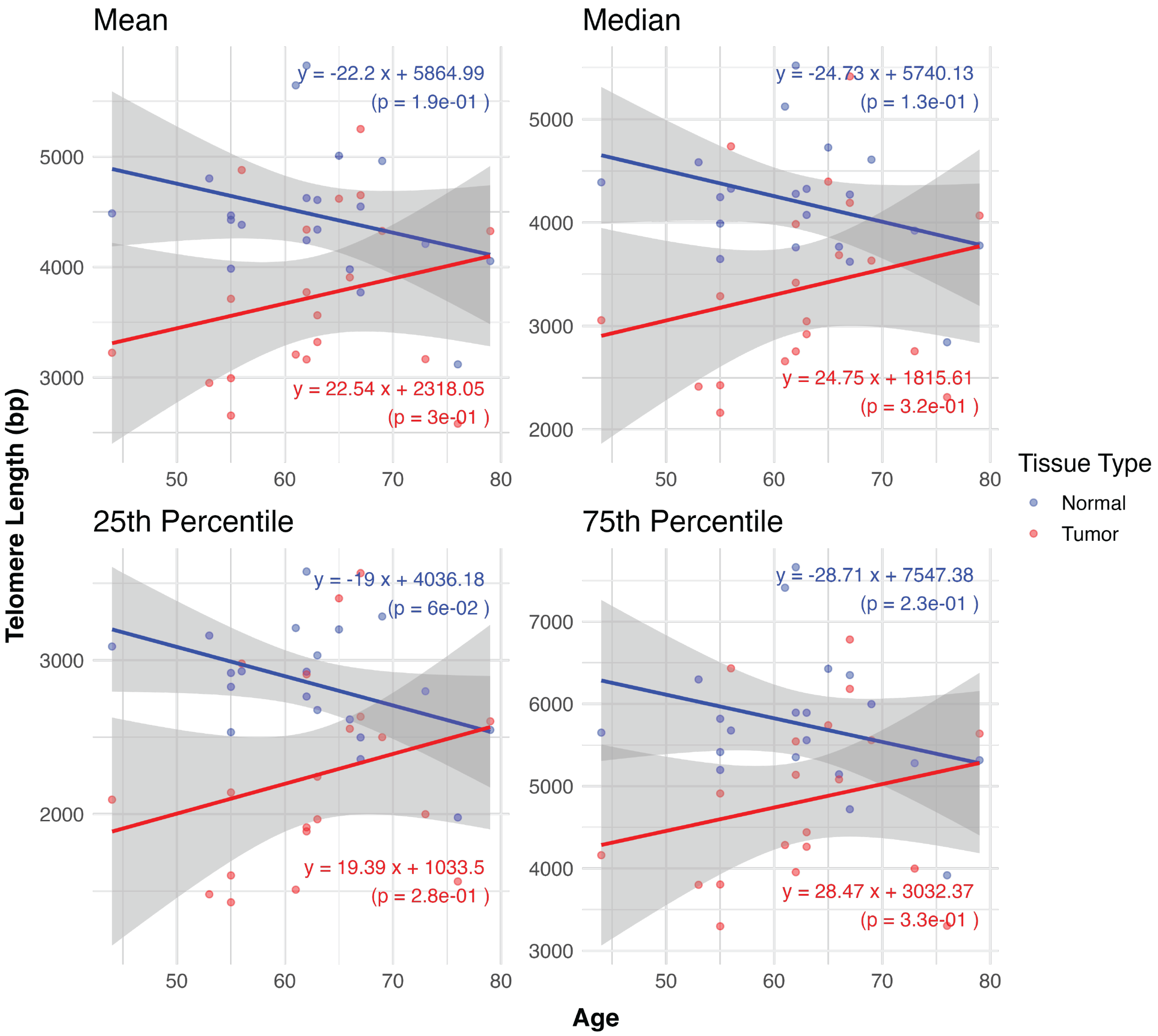

Supplementary Figure 6. Linear regression of telomere length summary statistics versus age from 20 patient-matched colorectal carcinomas (red, n=20 individuals) and normal colonic epithelia (blue, n=20 individuals). Companion to main-text Figure 4.

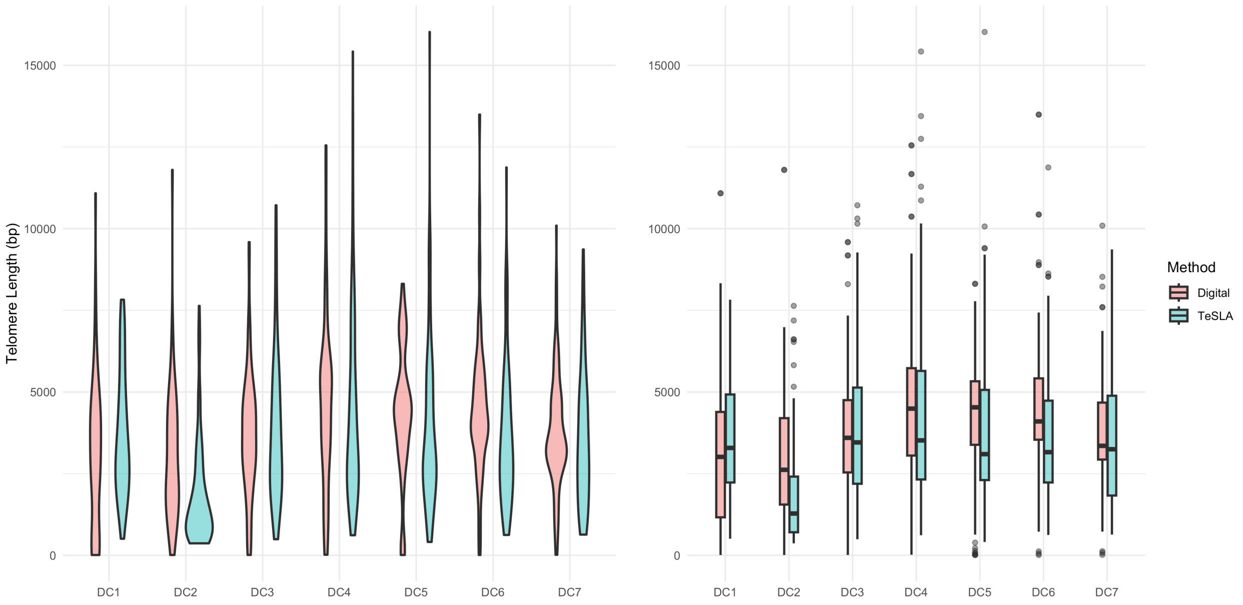

Supplementary Figure 7. Comparison of digital telomere measurements and in-gel measurements of TeSLA Southern blot bands from *RTEL1* variant cohort PBLs. Violin (left) and boxplot (right) of digital telomere measurements by long-read sequencing (red) and in-gel measurements of TeSLA Southern blot bands (blue) from *RTEL1* variant cohort PBLs. TeSLA measurements originally published in (*22*).

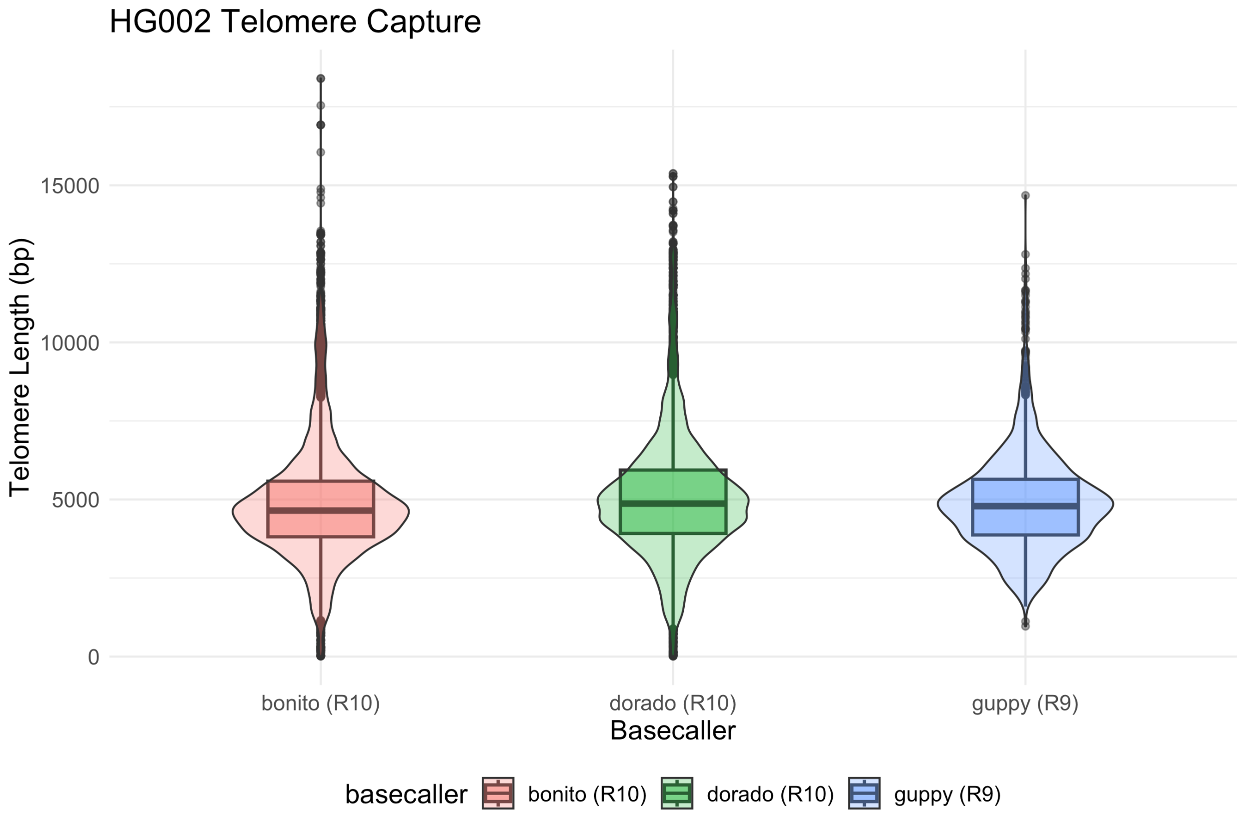

Supplementary Figure 8. HG002 digital telomere measurement and basecaller performance comparison. Telomere capture sequencing and digital measurement across two technical replicates (R9 or R10 sequencing chemistry) and three basecalling models. Bonito (custom ONT model HG002.k1, n=10465 telomeres) and dorado (v0.3.4, dna_r10.4.1_e8.2_400bps_sup@v4.2.0, n=10353,) models were used to basecall the same raw sequencing data. Guppy (v6.5.3, n=2122) R9 basecalls represent a technical replicate. Telomere measurement was performed with Telometer in all cases.

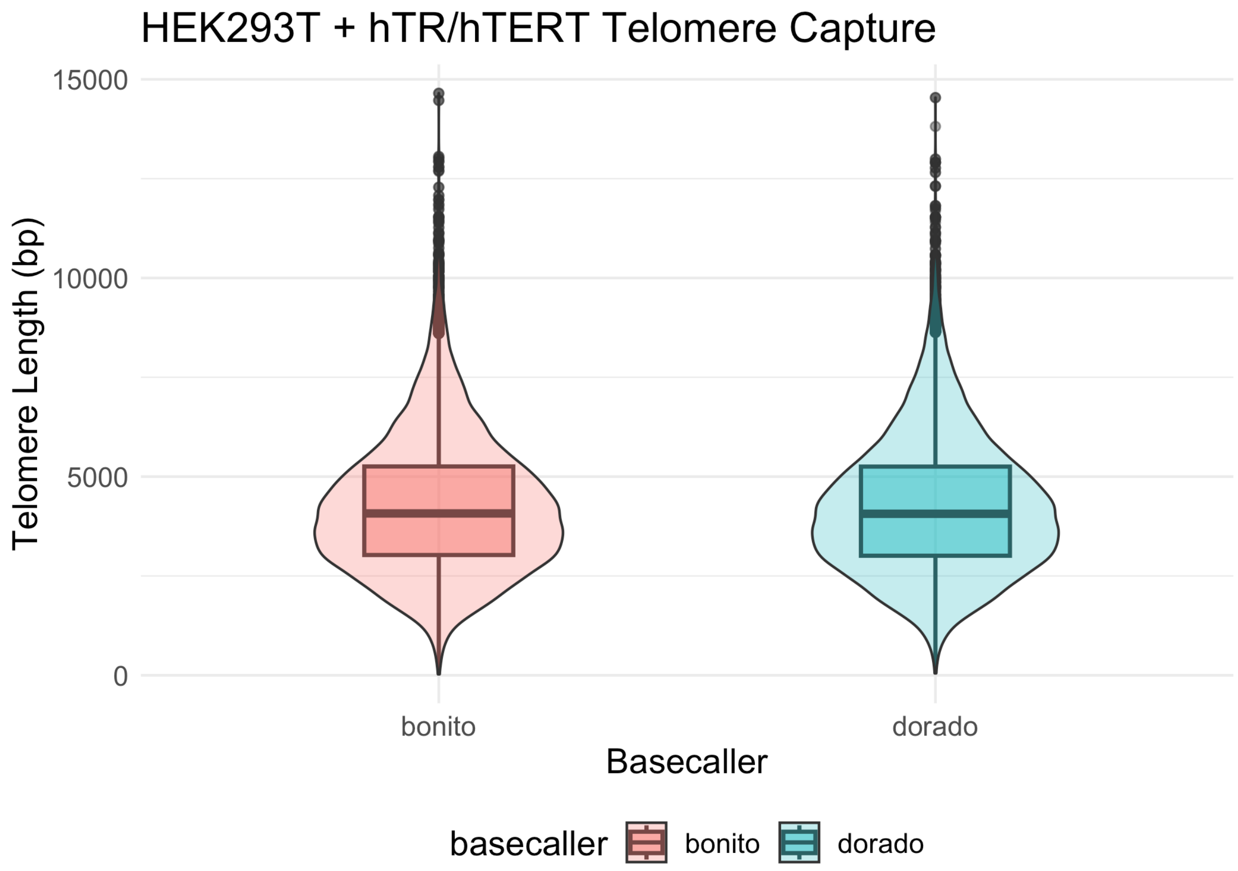

Supplementary Figure 9. HEK 293T digital telomere measurement and basecaller performance comparison. Telomere capture sequencing and digital measurement across two basecalling models. Bonito (custom ONT model HG002.k1, n=21402) and dorado (v0.3.4, dna_r10.4.1_e8.2_400bps_sup@v4.2.0, n=21282 telomeres,) models were used to basecall the same raw sequencing data. Telomere measurement was performed with Telometer in both cases.
