# Supplementary material for: Digital telomere measurement by long-read sequencing distinguishes healthy aging from disease
Source: Nat Commun. 2024 Jun 18;15:5148. doi: 10.1038/s41467-024-49007-4 (PMC11189511; doi:10.1038/s41467-024-49007-4)
Supplement: Supplementary file 3 — Description of Additional Supplementary Files [file 41467_2024_49007_MOESM3_ESM.pdf]

Description of Additional Supplementary Data

File: Supplementary Data 1
Description: Characteristics of healthy peripheral blood donors, RTEL1 mutants, and TB32

File: Supplementary Data 2
Description: Sample ID, age at biopsy, and telomere coverage of patient-matched benign colonic epithelia and tumor biopsies.

File: Supplementary Data 3
Description: Telomere Capture Oligo Sequences

File: Supplementary Data 4
Description: Binary classification model training and test results

File: Supplementary Data 5
Description: Telomere length summary statistics from single-source HG002 cells from two laboratories

.
