# Supplementary material for: Digital telomere measurement by long-read sequencing distinguishes healthy aging from disease
Source: Nat Commun. 2024 Jun 18;15:5148. doi: 10.1038/s41467-024-49007-4 (PMC11189511; doi:10.1038/s41467-024-49007-4)
Supplement: Supplementary file 10 — Source Data [file 41467_2024_49007_MOESM10_ESM.zip › Source Data Image File.docx]

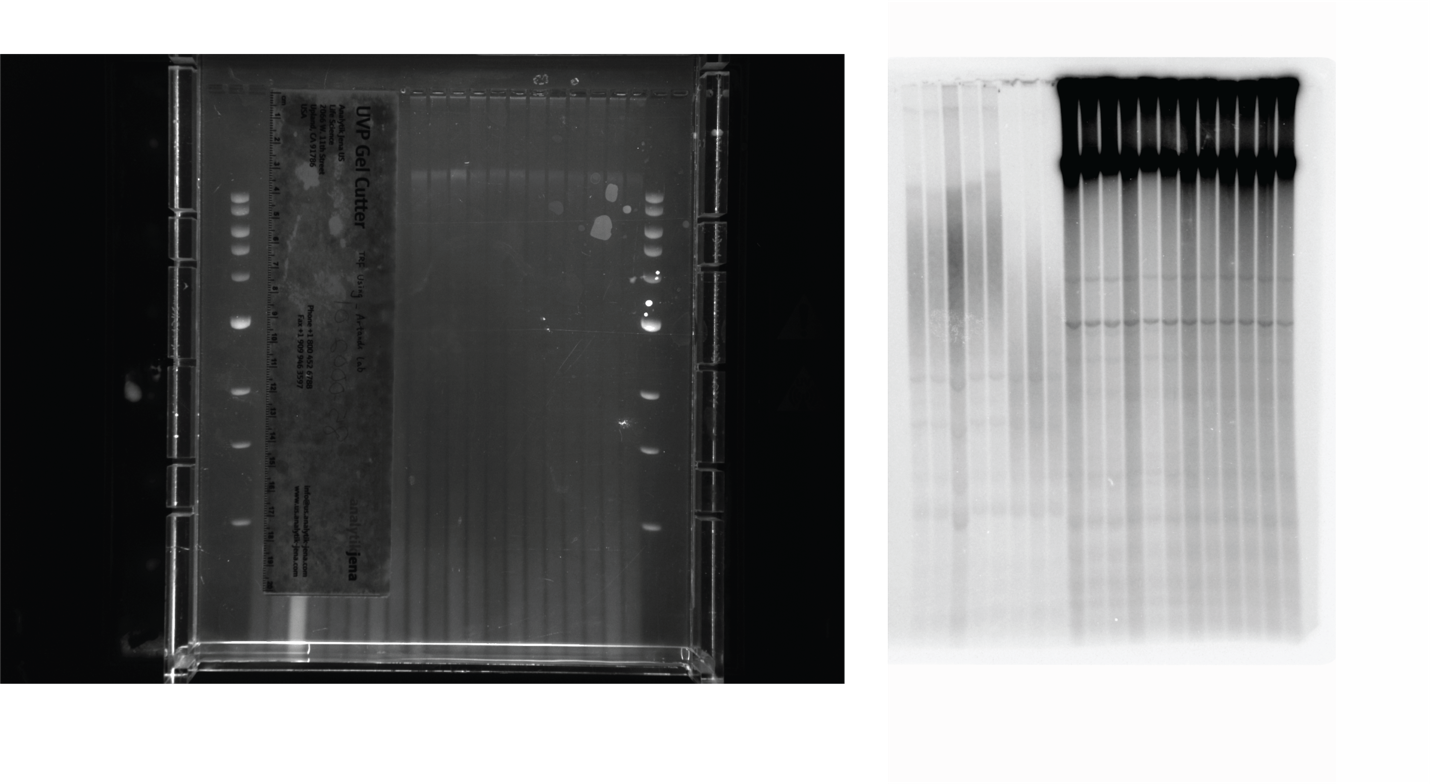


Source Image 1: hESC WT and PARN KO TRF Southern Blot. (Left) Ethidium Bromide Trans-UV image of TRF gel with UV-transluscent ruler superimposed. (Right) Phosphor-screen post-exposure image of TRF Southern blot. Samples to the right of lane 8 unrelated to this manuscript. Source for Figure 1J.


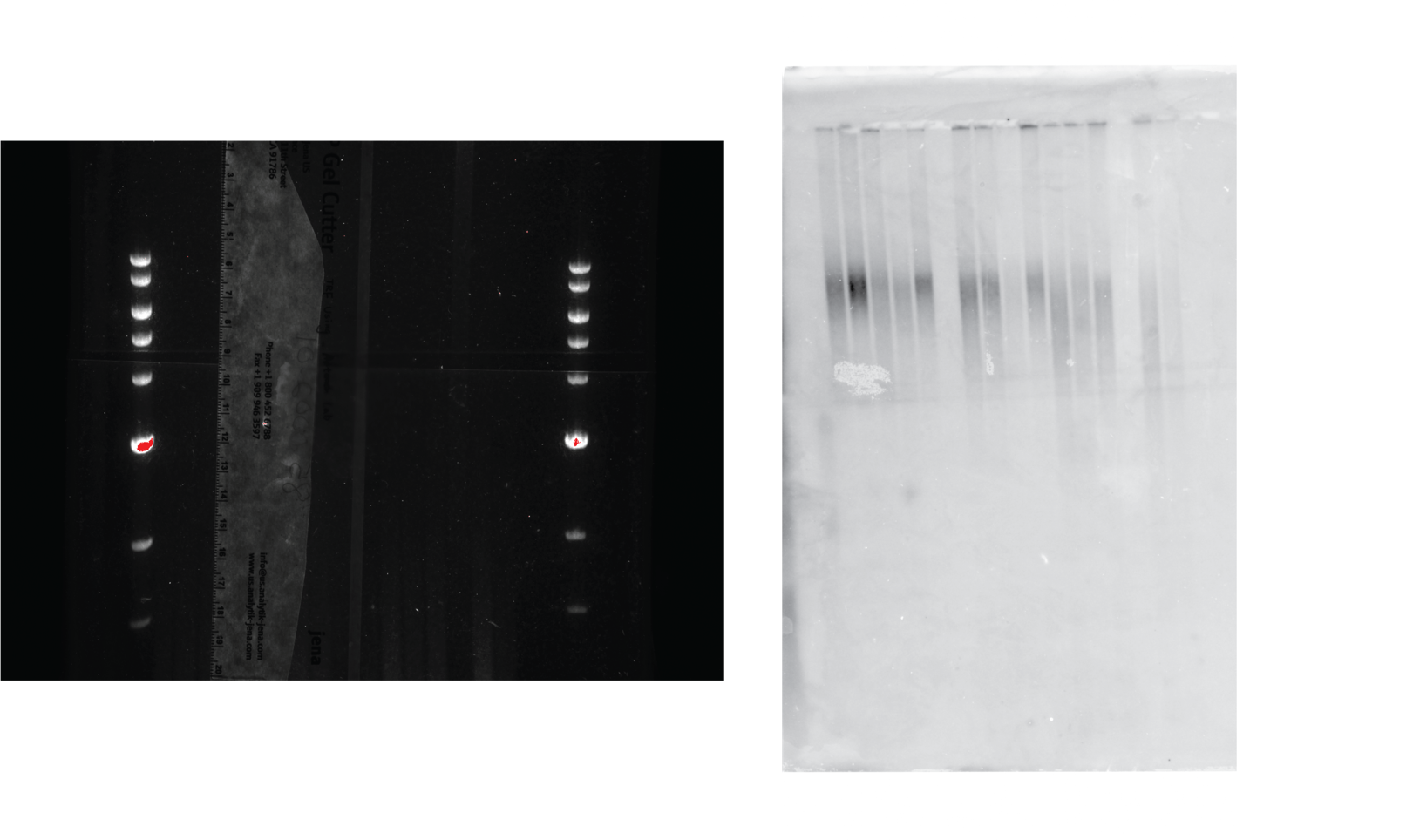


Source Image 2: Healthy Donor and TB32-B, TB32-M TRF Southern blot. (Left) Ethidium Bromide Trans-UV image of TRF gel with UV-transluscent ruler superimposed. (Right) Phosphor-screen post-exposure image of TRF Southern blot. Source for Figure 1D, 1E, Supplementary Figure 3.


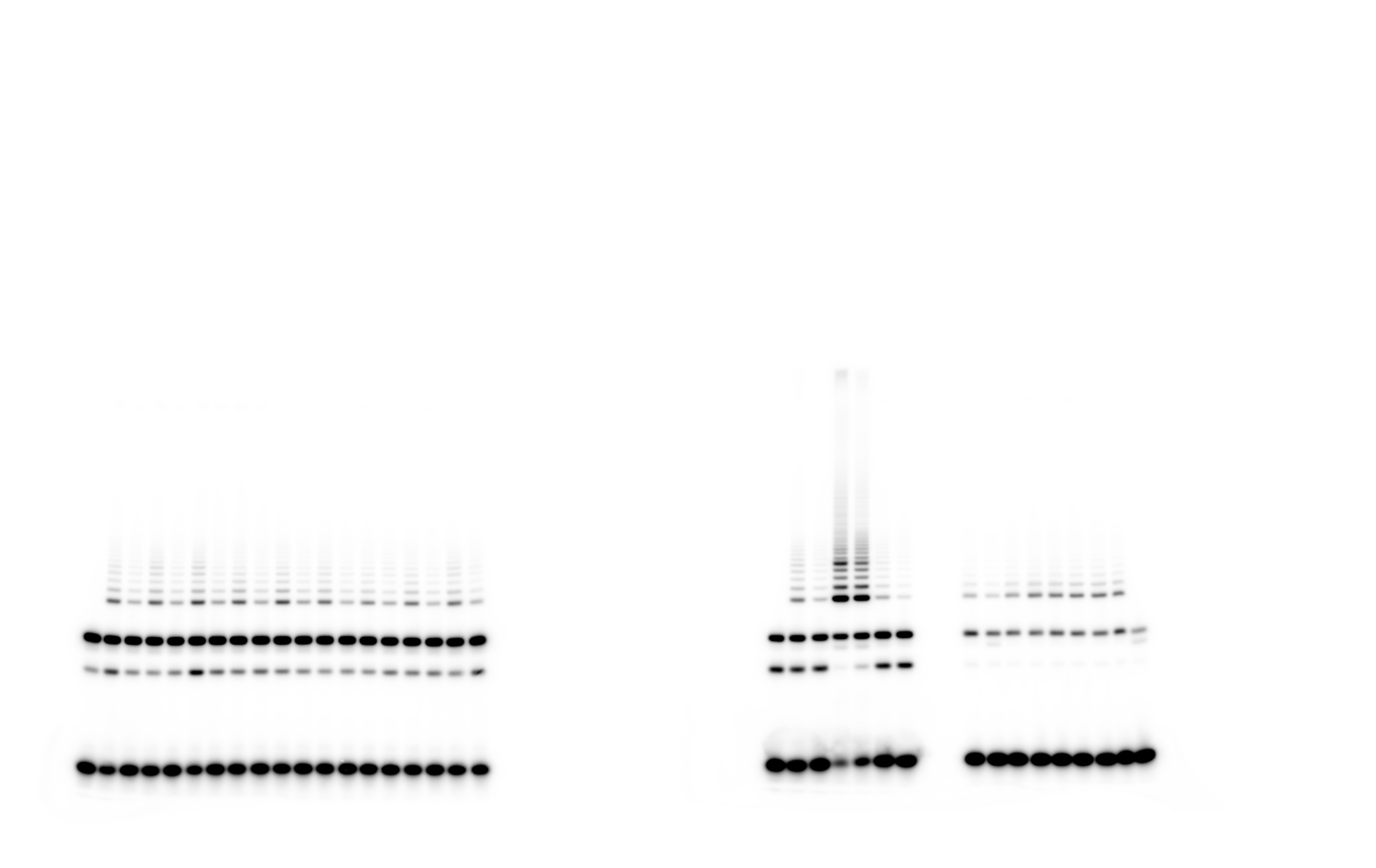


Source Image 3: TRAP gel of HEK293T + GFP, HEK293T + hTR/hTERT, HEK293T + TSQ/hTERT cell lysates among other samples unrelated to this manuscript. Source for Figure 2G (right gel, first 7 lanes).
